# Prognostic significance of chromosome 3 alterations determined by microsatellite analysis in uveal melanoma: a long-term follow-up study

**DOI:** 10.1038/bjc.2012.54

**Published:** 2012-02-21

**Authors:** S Thomas, C Pütter, S Weber, N Bornfeld, D R Lohmann, M Zeschnigk

**Affiliations:** 1 Department of Ophthalmology, University Hospital of Essen, Hufelandstrasse 55, Essen, Germany; 2 Institute for Medical Informatics, Biometry and Epidemiology, University of Duisburg-Essen, Essen, Germany; 3 Institute for Human Genetics, University Hospital of Essen, Hufelandstrasse 55, Essen, Germany

**Keywords:** uveal melanoma, eye, microsatellite analysis (MSA), prognosis, monosomy 3, partial monosomy 3

## Abstract

**Background:**

In uveal melanoma (UM), the most frequent primary intraocular tumour in adults, loss of one entire chromosome 3 (monosomy 3 (M3)) is observed in ∼50% of tumours and is significantly associated with metastatic disease. The strong association of metastatic disease with M3 offers the opportunity for molecular prognostic testing of UM patients.

**Methods:**

To re-evaluate M3 as prognostic marker in our clinical and laboratory setting and to determine the metastatic potential of rare tumours with partial M3, we performed a comprehensive study on 374 UM patients treated by enucleation in our clinic within 10 consecutive years, starting in 1998. Genotyping of all tumours was performed by microsatellite analysis.

**Results:**

Median follow-up time was 5.2 years. The disease-specific mortality rates (death by UM metastases) for tumours with disomy 3 (D3) and M3 were 13.2% and 75.1%, respectively. The disease-specific survival was worse when M3 was observed together with chromosome 8 alterations (*P*=0.020). Death of UM metastases was also observed in 12 patients (9%) with D3 tumours. The metastasising D3 tumours showed a larger basal tumour diameter (*P*=0.007), and were more frequently of mixed or epitheloid cell type (*P*<0.0001) than D3 tumours that did not metastasise. Mortality rate of tumours showing partial M3 (8.3%) was as low as that for tumours with D3.

**Conclusion:**

This shows that large tumours with disomy 3 have an increased risk to develop metastases. On the basis of these results, our clinic offers routine prognostic testing of UM patients by chromosome 3 typing.

Approximately 50% of uveal melanoma (UM) patients die of metastases, a proportion that has remained constant during the past century (Kujala *et al*, 2003). In 1996, Prescher *et al* discovered the strong association between the loss of an entire chromosome 3 (monosomy 3 (M3)) in the tumour and metastatic death of patients. This was later on confirmed by other studies, and is observed regardless of the genotyping technique used to determine the chromosome 3 status (Sisley *et al*, 1997; White *et al*, 1998; Scholes *et al*, 2003; Damato *et al*, 2010; Shields *et al*, 2011). Gain of chromosome 8q further modulates metastatic progression of M3 tumours but not of disomy 3 (D3) tumours. Global gene expression profiling (GEP) studies later revealed two distinct classes of UM tumours that are almost perfectly associated with chromosome 3 status and patient prognosis (Tschentscher *et al*, 2003; Onken *et al*, 2004). On the basis of more recent studies, GEP claims to be more accurate in predicting metastasis when compared with monosomy 3 detected by comparative genomic hybridisation or fluorescence *in situ* hybridisation (Worley *et al*, 2007).

The strong association of tumour classification with metastatic progression facilitates molecular prognostic testing of patients by either chromosome 3 testing or GEP, and studies have shown that the majority of patients want to know their risk of developing metastasis even though no treatment options currently exist (Beran *et al*, 2009; Damato and Coupland, 2009). Clinical application of such testing might emerge from the availability of adjuvant therapy for UM as only high-risk patients should be included in clinical trails.

Although the prognostic value of chromosome 3 loss in UM is well established, some tumours cannot be clearly classified into either high or low-risk groups based on chromosome 3 typing. These include tumours that show loss of only parts of chromosome 3, referred to as partial M3. Reports about the frequency of partial M3 tumours and prognosis of the patients are inconsistent (Damato *et al*, 2010; Abdel-Rahman *et al*, 2011; Shields *et al*, 2011). Tumour heterogeneity may also lead to equivocal results if chromosome 3 testing is done by multiplex ligation-dependent probe amplification (MLPA) or microsatellite analysis (MSA). Finally, in spite of the overall good prognosis a few patients with unequivocal disomy 3 in their tumours die from metastatic disease (Damato *et al*, 2010; Shields *et al*, 2011).

In 1997, we implemented MSA for chromosome 3 typing, replacing conventional cytogenetics and comparative genomic hybridisation methods. Although the prognostic accuracy of the GEP assay has been confirmed and GEP claims to be more accurate on prognostic testing than genotyping for monosomy 3, (Onken *et al*, 2004, 2010) in our hands GEP is more costly than genotyping methodologies and routine preparation of high-quality mRNA is demanding in clinical settings. To re-evaluate M3 as a prognostic marker in our clinical and laboratory setting, to determine the prognosis of patients with partial M3, and to better capture the prognostic value of an ambiguous chromosome 3 status, we performed a comprehensive retrospective study on 374 UM patients who were treated by enucleation in our clinic between January 1998 and December 2007, and for whom survival and genotyping data were available. Genotyping of all tumours was performed by MSA using eight chromosome 3 and four chromosome 8 markers (Tschentscher *et al*, 2000). We associated genetic and clinical data with death of patients caused by UM metastases. On the basis of the results of this study, we now perform routine MSA-based prognostic testing of UM patients who want to know about their metastatic risk.

## Patients and methods

### Patients

In this study, we present data from UM patients who had been treated at the Department of Ophthalmology of the University Hospital of Essen by primary enucleation without prior radiation between January 1998 and December 2007. We have included all patients in the study who signed the informed consent and from whom genotyping data and follow-up information are available. Enucleation was considered if the tumour height was >9 mm, if position of the tumour was juxtapapillary, and/or if patients refused eye bulb conserving therapies. Systemic clinical examination was performed routinely, including fundus photography and measurement of smallest and largest basal tumour diameter, and tumour thickness by B-scan and A-scan echography. The clinical data at initial examination included age, gender, affected eye, and tumour location (ciliary body involvement, choroideal). Tumour sampling was performed directly after enucleation by cutting the sclera from the contra lateral side and taking a part of the tumour tissue with a forceps. Tumour samples were snap-frozen in liquid nitrogen. For histopathological examination, the eye with the remaining tumour was formalin fixed and paraffin embedded. The clinical diagnosis of UM was histopathologically confirmed using sections stained for haematoxylin and eosin, HMB45, S100, Ki67, and Melan A. Each tumour was classified according to cell type (spindle, epitheloid, and mixed cell type) using the modified Callender system (McLean *et al*, 1982). Blood samples were obtained at the time of surgery.

The time and the cause of death were collected by contacting the registration and health offices, respectively. Death from UM metastatic disease was only assigned if so stated on the death certificate. This study was conducted in accordance with the Declaration of Helsinki and Good Clinical Practice Guidelines and was approved by the ethical committee of the University Hospital of Essen in 1996.

### Microsatellite analysis

DNA extraction and MSA have been slightly modified from Tschentscher *et al* (2000). Briefly, tumour material and peripheral blood were obtained at the time of surgery and stored at −80 °C and −20 °C, respectively. DNA was extracted from tumour tissue by a conventional phenol/chloroform procedure (Sambrook and Maniatis, 1989) and from blood using the FlexiGene Kit (Qiagen, Hilden, Germany). To remove melanin, which impairs the PCR, 6 *μ*g of tumour DNA were purified using the QIAamp tissue kit (Qiagen), according to the manufacturer's instructions and eluted twice with 150 *μ*l H_2_O. The DNA concentration was determined with the NanoDrop 2000 Spectrophotometer (Thermo Scientific, Wilmington, DE, USA). The following chromosome 3 and chromosome 8 polymorphic microsatellite loci were analysed: D3S3050-HEX, D3S1263-FAM, D3S1481-FAM, D3S2406-TET, D3S3045-FAM, D3S1744-TET, D3S2421-FAM, D3S1311-HEX, D8S1119-TET, D8S1132-FAM, D8S1128-TET, and D8S265-HEX. MSA primers were purchased from Eurogentec (Seraing, Belgium). Forward primers were linked to distinct fluorescent labels as indicated (HEX, FAM, TET) in order to enable single-lane analysis of markers from one chromosome. PCR was performed as follows: ∼40 ng template DNA were added to a 20-*μ*l reaction mixture containing 2 *μ*l 10 × Mastermix II (Applied Biosystems, Foster City, CA, USA), 1.25 mM each of deoxynucleotide triphosphate, 0.2 U *Taq* polymerase (Applied Biosystems), 8 pmol each primer pair, and T4gp32 (Q-BIOgene, Inc., Carlsbad, CA, USA) at a final concentration of 5 ng *μ*l^−1^. Cycling was performed in a GeneAmp PCR System 9600/9700 thermocycler (Applied Biosystems) with an initial denaturation step of 2 min at 94 °C, 35 cycles of denaturation (15 s at 94 °C), annealing (30 s at primer-specific temperature, 50–58 °C), extension (30 s at 72 °C), and a final extension step at 72 °C for 7 min. Aliquots of the PCR products were checked on an agarose gel to estimate the amount of product to be loaded on an ABI 3100 or 3130xl Genetic Analyser (Applied Biosystems). GeneScan and Genotyper software (Applied Biosystems) were used to evaluate the MSA data. To correct for the difference in amplification efficiency of different marker alleles, the ratio of allele peak areas (allele ratio (AR)) in the tumour was normalised against the peak ratio obtained in the corresponding DNA from blood. The genotype of a locus was assigned based on the calculated AR: ARs>2.5, loss of heterozygosity; AR<1.4, retention of heterozygosity. ARs in between were referred to as ‘allelic imbalance’ (AI). A tumour was classified as M3 or disomy 3 if all informative chromosome 3 markers showed loss of heterozygosity or retention of heterozygosity, respectively, and only one marker showing AI is permitted for a M3 or D3 classification. Partial M3 was assigned if at least one chromosome 3 marker showed loss of heterozygosity in a tumour with others showing retention of heterozygosity. Tumours in which more than one informative chromosome 3 marker shows AI were classified as AI. Chromosome 8 was assumed to be abnormal if at least one marker shows an AR⩾1.4.

### Statistical analyses

We used standard descriptive to display sample characteristics. Associations between clinical tumour characteristics and chromosome status were assessed either by non-parametric Wilcoxon–Mann–Whitney tests in the case of continuous variables or by generalised Fisher's exact test for categorical variables in 2 × m tables. To analyse time to event data, we used survival analysis. The time to death was calculated as the difference between the date of enucleation and metastases-related death. Patients, who were lost to follow-up or who died with unknown or other causes of death were censored. Melanoma-related survival probabilities were graphically displayed by the Kaplan Meier method (including a log-rank test to compare the curves globally). Univariate and multivariate cox regression analyses were used to evaluate the impact of the following covariates: sex, chromosome 3 status, tumour thickness, smallest basal tumour diameter, largest basal tumour diameter, chromosome 8 status, cell type, and ciliary body involvement. In the initial multivariate model, all main effects were investigated simultaneously. To avoid over fitting, a restricted model was assessed, including only those predictors with a *P*-value of 0.05 or lower in either the univariate or initial multivariate comparison. The assumption of proportional hazards was checked graphically and by a statistical test (Grambsch and Therneau, 1994) evidence for a deviation was observable for the comparison of M3 and D3 time-to-event curves (*P*=0.01). However, as no strong evidence for a deviation was observable globally across all groups, we decided to report the results of the cox model. Effect size estimators are presented with 95% confidence intervals (95% CI) and all reported *P*-values are explorative, two-sided and nominal (i.e., not adjusted for multiple testing). The level of significance *α* for each test was 0.05 (two-sided).

## Results

From January 1998 to December 2007 a total of 442 UM patients were treated by enucleation in our clinic. Tumour tissue, blood samples, and a signed informed consent was available from 403 patients. Genotyping of tumour samples using MSA on eight chromosome 3 markers and four chromosome 8 markers was successfully performed in 402 tumour samples. Follow-up data were available for 374 of the 402 patients. On the basis of the chromosome 3 MSA results, we classified the tumours into four groups: 128 tumours with disomy 3, 211 tumours with M3, 16 tumours with partial M3, and 19 tumours with AI (see Materials and Methods for description of AI). For each group the tumour characteristic and clinical features of patients are listed in Table 1. The average tumour thickness was 10.4 mm overall, 9.9 mm for disomy 3 tumours, 10.5 mm for M3 tumours, 11.3 mm for partial M3 tumours, and 11.3 mm for AI tumours. The mean largest basal diameter was 15.2 mm for all tumours and did not vary much between groups. Overall, the tumour was choroid in 260 cases (75%) and showed ciliary body involvement in 94 cases (25%). Patients with M3 tumours were on average older (66.5 years) than patients with D3 tumours (61 years; *P*=0.001).

**Figure 1:**
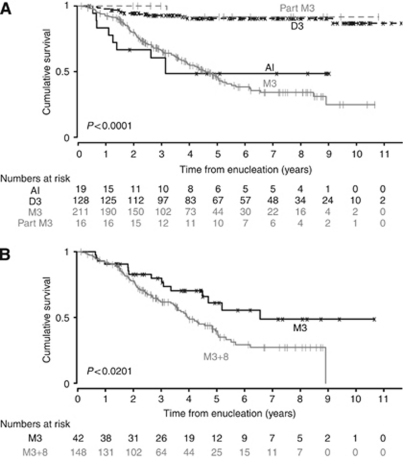
Kaplan–Meier survival curves showing disease-specific cumulative survival according to chromosome 3 status (**A**) and according to chromosome 3 and 8 status (**B**). AI, patients showing allelic imbalance of chromosome 3 in the tumour; part M3, tumours with partial monosomy 3; D3, tumours with disomy 3; M3, tumours with monosomy 3; M3+8, tumours showing monosomy 3 and chromosome 8 alterations.

Follow-up data were available from 374 patients with a median follow-up time of 5.2 years. A total of 195 of these patients died and the diagnosed cause of death was metastasis from UM in 124 patients (63%), and second cancer in 10 patients. In the remaining 61 patients, the cause of death was either non-neoplastic or unknown because we were unable to obtain information. Only death by UM metastases were considered as events whereas all other death events were coded as censored. For this outcome, death by UM metastases, disease-specific survival curves for each of the four classes of chromosome 3 alteration (D3, M3, AI, and partial M3) are shown in Figure 1. Pairwise comparisons were made and except for AI *vs* M3 and D3 *vs* part M3 all pairwise comparisons had *P*-values <0.01.

In our patient cohort, loss of one chromosome 3 in the tumour was strongly associated with metastatic death of patients, and survival in the M3 group was even worse when additional chromosome 8 alterations were present as observed in previous studies (Sisley *et al*, 1997; White *et al*, 1998). UM-related survival was similarly low in patients with AI in their tumours as compared with M3 carriers. In multivariate analysis, a significant association with disease-related survival was also observed for the largest basal diameter (Table 2).

**Figure 2:**
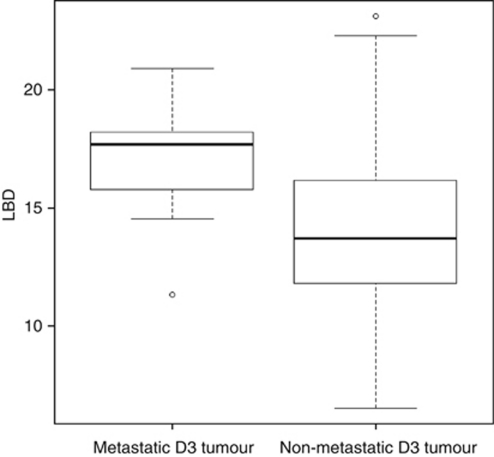
Box plot presenting the distribution of largest basal diameter (LBD) of tumours with disomy 3, grouped according to their metastatic progression. Rectangle shows the interquartile range (IQR). The median is depicted by a solid line. Whiskers extend to the most extreme value within 1.5 × IQR above or below the box. Open circle: outlier that falls outside the inner fence.

Retention of both chromosomes 3 in a tumour is associated with a good overall prognosis, whereas a total of 12 of the 128 patients with disomy 3 in their tumour died of metastasis. To identify markers that might facilitate identification of the rare high-risk tumours within the D3 tumour class, we compared the clinical and genetic features of non-metastasising D3 tumours with metastasising D3 tumours (Table 3). We found a significant association for the cell type (*P*<0.0001) as 58% of metastasising D3 tumours had an epitheloid or mixed cell type, which was found in only 8% of non-metastasising D3 tumours (Table 3). A significant difference was also observed in largest basal tumour diameter (*P*=0.007), which was smaller in non-metastasising D3 tumours (13.97 mm) than in metastasising tumours (16.96 mm; Figure 2). A similar trend was observed for the largest basal diameter in tumours with M3.

Interestingly, a low mortality rate (8.3%) was found for tumours showing partial M3 (Figure 1) irrespective of which of the eight chromosome 3 markers showed loss of heterozygosity (data not shown). Partial M3 is assigned if at least one informative marker shows loss (AR>2.5) and the remaining informative marker(s) show retention of heterozygosity (AR<1.3). Of 16 patients with tumours classified as partial M3, which had a median follow-up time of 5.5 years, only one developed metastases. The other 15 patients were still alive at the close of study.

## Discussion

In this paper, we present long-term follow-up data on 374 UM patients that were all treated by enucleation. We associated disease-related death of patients with the chromosome 3 and 8 status of their tumours as determined by MSA. The results of the follow-up study confirmed the long-standing prognostic value of M3 in UM in our test assembly. As we included only enucleated tumours, the results are limited mainly to patients with large or fairly large tumours, because smaller ones are routinely treated with eye conserving methods.

In our experimental setting using MSA for genotyping, a small subset of tumours shows AI (defined if at least two informative chromosome 3 markers show an AR between 1.4 and 2.5). A plausible explanation for this outcome is that tumours with AI are composed of cells with M3 and D3. Metastatic progression from a mixed tumour might then originate from the cells with M3 irrespective of the nature and portion of cells with disomy 3 which might be tumour cells or non-tumour cells. A similar observation was made by Damato *et al* using MLPA for chromosome 3 typing (Damato and Coupland, 2009; Damato *et al*, 2010). They found that patients with ‘borderline’ or ‘equivocal abnormality,’ of chromosome 3 are also more likely to die from metastases and suggested that these MLPA results are due to a heterogeneous mixture of melanoma cells. Therefore, AI as determined by MSA might be synonymous with ‘borderline’ or ‘equivocal’ loss as defined by MLPA. These two lines of evidence support the idea that patients with AI tumours defined by MSA should be given a poor prognosis.

In our cohort, partial M3 was found in tumours of only 16 patients (4%), which is at the lower end of partial M3 frequencies reported in other studies (0–48% Damato *et al*, 2010; Abdel-Rahman *et al*, 2011; Shields *et al*, 2011). An unexpected finding of this study was that only 1 of these 16 patients died of metastases, whereas the other 15 patients were still alive at the close of the study. In other studies, mortality rates of patients with partial M3 are much higher (Damato *et al*, 2010; Shields *et al*, 2011). Various methodologies such as MLPA, MSA, conventional cytogenetics, and comparative genomic hybridisation, as well as different classification criteria, have been used, making a comparison of these studies difficult. In the study by Shields *et al*, the analysed samples were from smaller tumours obtained by fine-needle aspiration biopsy, which might explain the observed divergence regarding partial M3. However, the tumours analysed in the study by Damato *et al* (2010) were exclusively obtained by enucleation or local resection thus covering the larger tumours. To improve the utility of routine prognostic testing by chromosome 3 typing a combined effort should be made to resolve the risk of metastasis associated with partial M3. In the past, partial chromosome 3 deletions in UMs have been mapped to obtain positional information on putative tumour-suppressor genes (Parrella *et al*, 1999; Tschentscher *et al*, 2001; Cross *et al*, 2006). However, in most studies systematic disease-specific survival analyses of the patients have not been performed. Our observation that partial M3 tumours rarely metastasise does not therefore support a major role for genes affected by the partial deletions in metastatic progression of UM.

In spite of the overall good prognosis for disomy 3, 9% of all patients (12 patients) with disomy 3 in their tumour (D3met tumours) died from metastasis, a percentage similar to that found in other studies using chromosome 3 testing (Damato and Coupland, 2009). It has been proposed that this could be explained by mis-sampling of cells with a normal chromosome 3 status from tumours otherwise composed of tumour cells with M3, or by misclassification of UM that have a partial deletion of chromosome 3 (Damato and Coupland, 2009). However, in our study, five of the D3met tumours showed chromosome 8 alterations, excluding the possibility of sampling of normal cells in at least these samples. As partial M3 tumours rarely metastasise, it seems unlikely that D3met tumours are mis-classified partial M3 tumours. Interestingly, we found a statistically significant association of metastatic progression with cell type and largest basal tumour diameter only when confining the analysis on the class of tumours with disomy 3. Both features are long known to predict metastatic disease in UM patients (McLean *et al*, 1982; Damato and Coupland, 2009). However, our data suggest that these features might be of particular relevance for the D3 class of tumours. Although the statistically significant association is weakened by the relative small number of D3 tumours showing metastatic progression, our conclusion is further supported by clinical data on seventeen D3met tumours presented in a different study (Lake *et al*, 2010). In this study a similar trend towards large basal diameter and cell type of these D3met tumours is suggested. Therefore, patients with large D3 tumours (>15 mm, Figure 2) or D3 tumours composed of spindle or mixed cell types do not have a favourable prognosis. The association of largest basal diameter with metastatic progression of the tumour further suggests that D3 tumours might acquire metastatic potential late during tumour progression. In the M3 tumour class this association is much less pronounced, suggesting that M3 tumour cells acquire their metastatic potential at early stages.

In summary, this study confirms the value of chromosome 3 typing by MSA for prognostic testing of UM patients in our clinical and laboratory setting. In addition we show that among patients with D3 tumours those with large tumours or tumours composed of spindle or mixed cell types have an increased risk to develop metastases. On the basis of these results, our clinic offers routine prognostic testing of UM patients by chromosome 3 typing.

## Figures and Tables

**Table 1:** Tumour features and clinical data of patients included in the study

	**All tumours (*n*=374)**	**D3 (*n*=128)**	**M3 (*n*=211)**	**AI (*n*=19)**	**Part M3 (*n*=16)**
Age at enucleation (years)	64.2±12.9	61.6±13.5	66.5±12.1	61.7±12.8	57.9±12.4
Male	209 (56%)	81 (63%)	103 (49%)	12 (63%)	13 (81%)
Female	161 (43%)	45 (35%)	106 (50%)	7 (37%)	3 (19%)
					
Tumour thickness (mm)	10.4±2.7	9.9±3.2	10.5±2.5	11.3±1.6	11.3±2.3
Smallest basal diameter (mm)	12.1±4.9	11.8±4.4	12.2±5.2	11.5±7.1	12.9±3.2
Largest basal diameter (mm)	15.2±3.7	14.3±3.5	15.7±3.6	16.9±3.8	15.5±3.8
					
Chromosome 8 normal	103 (28%)	46 (36%)	42 (20%)	5 (26%)	2 (13%)
Chromosome 8 alteration	220 (59%)	54 (42%)	148 (70%)	13 (68%)	13 (81%)
					
*Cell type*					
Epitheloid	5 (1%)	1 (1%)	3 (1%)	1 (5%)	0 (0%)
Mixed	92 (25%)	15 (12%)	68 (32%)	8 (42%)	1 (6%)
Spindle	271 (72%)	109 (85%)	138 (65%)	10 (53%)	14 (88%)
					
*Ciliary body involvement*					
No	260 (70%)	94 (73%)	139 (66%)	12 (63%)	15 (94%)
Yes	94 (25%)	23 (18%)	65 (31%)	5 (26%)	1 (6%)
					
*Extraocular extension*					
No	331 (89%)	113 (88%)	184 (87%)	19 (100%)	15 (94%)
Unclear	14 (4%)	8 (6%)	6 (3%)	0 (0%)	0 (0%)
Yes	29 (8%)	7 (5%)	21 (10%)	0 (0%)	1 (6%)

Abbreviations: AI=allelic imbalance; D3=disomy 3; M3=monosomy 3.

Reported are total counts (percentages in parentheses) and for continuous variables mean±standard deviation.

**Table 2:** Cox regression analysis in patients with uveal melanoma (*n*=374)

			**Multivariate**			
	**Univariate**		**Initial**		**Restricted**	
**Covariable**	**HR (95% CI)**	***P*-value**	**HR (95% CI)**	***P*-value**	**HR (95% CI)**	***P*-value**
Chr 3 status	—	—	—	—	—	—
Disomy 3	1	—	1	—	1	—
Allelic imbalance	7.74 (3.25–18.43)	<0.001	5.92 (1.92–18.25)	0.001	4.56 (1.52–13.67)	0.007
M3	8.10 (4.42–14.84)	<0.001	8.06 (3.58–18.16)	<0.001	7.16 (3.32–15.41)	<0.001
Part M3	0.64 (0.08–4.95)	0.672	0.82 (0.10–6.74)	0.850	0.75 (0.09–6.06)	0.787
						
*Gender*	—	—	—	—	—	—
Male	1	—	1	—	—	—
Female	1.12 (0.79–1.60)	0.530	0.70 (0.44–1.12)	0.139	—	—
						
Tumour thickness (mm)	1.02 (0.96–1.09)	0.529	0.91 (0.82–1.01)	0.081	—	—
Smallest basal diameter (mm)	1.06 (1.01–1.11)	0.021	1.03 (0.97–1.08)	0.345	—	—
Largest basal diameter (mm)	1.14 (1.08–1.20)	<0.001	1.10 (1.02–1.19)	0.014	1.09 (1.03–1.16)	0.003
						
*Chr 8 alteration*	—	—	—	—	—	—
No	1	—	1	—	1	—
Yes	2.08 (1.31–3.29)	0.002	1.72 (1.00–2.98)	0.051	1.47 (0.88–2.46)	0.137
						
*Cell type*	—	—	—	—	—	—
Epitheloid	1	—	1	—	1	—
Mixed	0.49 (0.15–1.60)	0.240	0.42 (0.12–1.49)	0.180	0.57 (0.17–1.96)	0.376
Spindle	0.16 (0.05–0.52)	0.002	0.25 (0.07–0.88)	0.031	0.37 (0.11–1.27)	0.114
						
*Ciliary body involvement*	—	—	—	—	—	—
No	1	—	1	—	—	—
Yes	1.22 (0.82–1.83)	0.332	1.14 (0.70–1.86)	0.585	—	—

Abbreviations: CI=confidence interval; HR=hazard ratio; M3=monosomy 3.

**Table 3:** Tumour characteristics and clinical features of patients with M3 and D3 tumours stratified according to their metastatic progression

	**M3**			**D3**		
	**Metastatic death**	**No metastases**	***P*-value**	**Metastatic death**	**No metastases**	***P*-value**
*N*	102	109		12	116	
Age at enucleation (years)	65.22±10.76	67.71±13.19	0.047	61.09±11.15	61.67±13.77	0.867
						
*Gender*						
Male	52	51		8	73	
Female	50	56	0.679	4	41	1
						
*Tumour size*						
Tumour thickness (mm)	10.61±2.40	10.48±2.54	0.826	9.41±3.46	9.99±3.21	0.750
Smallest basal diameter (mm)	12.82±5.19	11.57±5.14	0.139	14.04±6.05	11.62±4.22	0.042
Largest basal diameter (mm)	16.29±3.77	15.13±3.44	0.054	16.96±2.70	13.97±3.41	0.007
						
*Chr 8 alteration*						
No	15	27		5	49	
Yes	77	71	0.080	4	42	1
						
*Cell type*						
Epitheloid	2	1		1	0	
Mixed	38	30		6	9	
Spindle	61	77	0.238	5	104	<0.0001
						
*Ciliary body involvement*						
No	71	68		8	86	
Yes	27	38	0.231	3	20	0.450

Abbreviations: D3=disomy 3; M3=monosomy 3.
